# Locally Advanced Colorectal Cancer: True Peritoneal Tumor Penetration is Associated with Peritoneal Metastases

**DOI:** 10.1245/s10434-017-6037-6

**Published:** 2017-10-26

**Authors:** Charlotte E. L. Klaver, Nadine C. M. van Huijgevoort, Anthony de Buck van Overstraeten, Albert M. Wolthuis, Pieter J. Tanis, Jarmila D. W. van der Bilt, Xavier Sagaert, André D’Hoore

**Affiliations:** 1 Department of Surgery, Academic Medical Centre, University of Amsterdam, Amsterdam, The Netherlands; 2 Department of Pathology, UZ Leuven, Louvain, Belgium; 3 Department of Abdominal Surgery, UZ Leuven, KU Leuven, Louvain, Belgium; 4 Department of Surgery, Flevoziekenhuis, Almere, The Netherlands

## Abstract

**Background:**

Findings show T4 colorectal cancer (CRC) to be a risk factor for the development of peritoneal metastases (PM). Heterogeneity regarding peritoneal involvement of T4 tumors might explain the wide range of reported PM incidences (8–50%). Hyperplastic and mesothelial inflammatory reactions complicate evaluation of the exact primary tumor involvement of the peritoneal layer. This retrospective cohort study aimed to assess the association between either inflammatory peritoneal reaction or peritoneal involvement of the primary tumor and the risk of PM.

**Methods:**

Since 2010, pathologists at UZ Leuven have systematically categorized peritoneal involvement in peritoneal reaction with tumor less than 1 mm from the peritoneal surface or true peritoneal penetration. All patients undergoing resection of CRC between January 2010 and July 2013 who fulfilled either of these pathologic criteria were included in this study.

**Results:**

The study enrolled 159 CRC patients. Peritoneal reaction with tumor less than 1 mm from the peritoneal surface was present in 43 patients and true peritoneal penetration in 116 patients. Overall, 29 patients (18%) had synchronous PM, and 30 patients (23%) had metachronous PM. In the multivariable analysis, true peritoneal penetration, in contrast to peritoneal reaction with tumor less than 1 mm from the peritoneum, was associated with greater risk of PM (odds ratio [OR], 2.518; range, 1.038–6.111; *p* = 0.041) and lymph node involvement (N1: OR, 1.572; range, 0.651–3.797 vs N2: OR, 4.046; range, 1.549–10.569; *p* = 0.014).

**Conclusion:**

Histologically confirmed true peritoneal penetration by CRC, rather than inflammatory peritoneal reaction constitutes a high risk for PM. With evolving treatment strategies that aim to treat PM in an earlier phase, identification of high-risk patients becomes highly important clinically.

**Electronic supplementary material:**

The online version of this article (doi:10.1245/s10434-017-6037-6) contains supplementary material, which is available to authorized users.

Locally advanced (stage T4) colorectal cancer (CRC) is subdivided into T4a (penetration of the visceral peritoneum) and T4b (adjacent organ invasion) (Table S1, tumor-node-metastasis [TNM]7) and constitutes a risk factor for the development of peritoneal metastases (PM).^1^–^5^ It is hypothesized that PM development is a consequence of malignant cells detached from the primary T4 tumor entering the peritoneal cavity. Subsequently, these free cells attach to the peritoneal surface and progress into PM.^6^,^7^

The reported risks for the development of PM in T4 CRC vary widely, from 8 to 50%. This variation can be partly explained by the heterogeneity of T4 tumors with respect to local peritoneal involvement. Hyperplastic and mesothelial inflammatory reactions of the peritoneum often complicate evaluation of the exact tumor involvement of the very thin peritoneal layer. As a consequence, it cannot be clearly determined whether a certain group of CRC tumors should be classified as T3 or T4.

In 2002, a narrow definition of T4 was determined for TNM6,^8^ encompassing tumors that perforate the visceral peritoneum. In 2006, Compton^9^ proposed a wider definition of T4 that also included hyperplastic and inflammatory reactions of the peritoneum. Since then, different interpretations of pT4 among pathologists have been used.

Conceivably, true tumor penetration of the peritoneum would result in the highest risk for PM. Shepherd et al.^10^,^11^ earlier showed the prognostic importance of subcategorizing tumors based on the extent of local peritoneal involvement (LPI; Table S2), distinguishing hyperplastic reactions, mesothelial inflammation, and true penetration of the peritoneum. However, few patient data exist on the risk for PM in these subcategories, probably due to restricted therapeutic consequences.

Currently, attention for T4 stage colon cancer is growing, with studies aiming to prevent the development of PM by prophylactic hyperthermic intraperitoneal chemotherapy (HIPEC)^12^ or to detect PM at an early but still curable stage by second-look surgery. This study aimed to investigate the association between true peritoneal penetration of the primary tumor, in contrast to peritoneal reaction with tumor very close to the peritoneum (within 1 mm), and the development of PM in CRC.

## Methods

### Patients

Patients were selected from a CRC database of the University Hospital Leuven (UZ Leuven). Since 2010, pathologists of UZ Leuven choose to define pT4 as true peritoneal tumor penetration or peritoneal hyperplastic of inflammatory reaction with tumor less than 1 mm from the peritoneum, in line with the considerations of Compton.^9^ They have systematically subclassified locally advanced tumors as true peritoneal tumor penetration, as peritoneal reaction with tumor less than 1 mm from the peritoneum, or as tumor without peritoneal involvement (i.e., tumors invading adjacent retroperitoneal organs or structures).

The current study enrolled all patients with an intraperitoneally located primary CRC (above the peritoneal reflection) undergoing resection between January 2010 and July 2013 with either peritoneal tumor penetration or peritoneal reaction. If the peritoneal involvement was not systematically scored in the pathology report, the patient was excluded from the study.

### Pathology

Per colorectal cancer specimen, one formalin-fixed, paraffin-embedded (FFPE) block per centimeter of tumor was collected (at random locations). From all areas with macroscopic suspicion of deepest ingrowth, a separate block was collected. Between two and five slides were retrieved per block to determine deepest penetration. In case a tumor within 1 mm of the peritoneal surface was detected, at least three deeper slides were retrieved from the relevant block to detect potential true tumor penetration. In case of hesitation, more material was collected. True tumor penetration was defined as tumor cells at the peritoneal surface or free tumor cells on the peritoneum with underlying ulceration of the peritoneum.

### Variables

Synchronous PM was defined as the presence of PM at the time of CRC diagnosis. Metachronous PM was defined as PM diagnosed during routine follow-up assessment by any methods or combination of methods such as imaging, re-laparotomy, or both.

Perforation refers to tumor or bowel perforation or suspected perforation based on clinical or intraoperative findings. Radicality of resection is subcategorized into three groups: R0 (radical resection with >1-mm tumor-free margin), R1 (microscopically nonradical resection involving ≤1-mm margin), and R2 (macroscopically nonradical resection).

### Statistical Analysis

Baseline characteristics are expressed as counts and percentages. Differences in baseline characteristics between groups were analyzed using a Chi square test or Fisher’s exact test. For normally distributed continuous variables, mean and standard deviation are given, and for non-normally distributed continuous variables, median and interquartile range (IQR) are reported.

Using logistic regression, independent factors associated with PM were identified. For the purpose of this analysis, a combined variable of synchronous and metachronous PM was used. Patients lost to follow-up evaluation without signs of PM within 3 years were categorized as “unknown.” Variables that were significant independent predictive factors in the univariable analysis (*p* ≤ 0.10) were included in the multivariable regression analysis. In the multivariable analysis, multicollinearity was assessed.

Association with metachronous PM was evaluated using Kaplan–Meier curves and Cox regression. Kaplan–Meier curves were truncated when numbers at risk became less than one-third of the starting group. For this analysis, patients with synchronous PM were excluded. Again, factors significantly associated with metachronous PM (*p* ≤ 0.10) in the univariable analysis were included in the multivariable analysis. In the multivariable analysis, a *p* value of 0.05 or lower was considered statistically significant. Because patients with radical resected primary T4 tumors that had no synchronous peritoneal or distant metastases are considered potentially eligible for studies on prophylactic therapy, the Cox regression analysis was repeated for this subgroup. Statistical analyses were performed using PASW Statistics, version 22 (SPSS Inc., Chicago, IL, USA).

## Results

### Patients

Between January 2010 and July 2013, 973 patients underwent surgery for primary CRC at UZ Leuven. Locally advanced tumor was diagnosed for 183 of these patients, fulfilling one of the histologic criteria used by the pathologists of UZ Leuven since 2010. The study excluded 17 patients because the pathologic peritoneal involvement was not systematically scored. Another seven patients were excluded because the primary tumor did not involve the peritoneum. Of the 159 patients included in this analysis, 43 had “peritoneal reaction with tumor less than 1 mm from the peritoneal surface” and 116 had “true peritoneal tumor penetration” (Fig. S1).

The mean age of the patients was 69 ± 13 years. For the majority of the patients, the diagnosed tumor was located in the recto sigmoid (44%) or the ascending colon (28%). All the patients had a diagnosis of adenocarcinoma, with a mucinous component in 26% of the patients. Of all the patients, 75% had surgery in the elective setting, and 42% underwent an initially laparoscopic resection. At time of diagnosis, 62% of the patients had lymph node metastases, and 36% had distant metastases. The median follow-up period was 35 months (IQR, 15–48 months), and the median overall survival time was 19 months (IQR, 11–31 months). Baseline characteristics were comparable between the two histologic subgroups (Table 1).

**Table 1:** Patient, tumor, and surgical characteristics

	<1 mm (*n* = 43)		Penetration (*n* = 116)		*p* value
	Counts	%	Counts	%	
Gender					
Male	21	49	53	46	0.724
Female	22	51	63	54	
Age (years)					
<60	7	16	25	22	0.281
60–70	14	33	33	28	
70–80	8	19	34	29	
>80	14	33	24	21	
ASA					
1–2	26	68	77	73	0.563
3–4	12	32	28	27	
Location					
Colon	40	93	106	91	
Rectum	3	7	10	9	
Right versus left location					
Right colon	15	41	34	34	0.138
Transverse colon	6	16	7	7	
Left colon/rectum	16	43	59	59	
Emergency setting	7	17	31	27	0.183
Approach					
Laparoscopic	15	35	31	27	0.395
Converted	7	16	14	12	
Open	21	49	70	61	
HIPEC	1	2.3	10	8.6	0.165
Perforation	2	4.7	15	13	0.133
Lymph node involvement					
N0	21	49	39	34	0.136
N1	15	35	42	37	
N2	7	16	34	30	
Synchronous distant metastases other than PM	6	14	31	27	0.091
Neoadjuvant therapy					
None	37	86	102	90	0.745
Chemotherapy	4	9.3	7	7.0	
(Chemo)radiotherapy	2	4.7	4	3.5	
Adjuvant chemotherapy	22	54	77	70	0.060
Mucinous (component)	9	21	32	28	0.394
Grade					
Poorly	7	17	27	25	0.442
Moderately	31	74	68	63	
Well	4	9.5	13	12	
Radicality					
R0	43	100	107	93	0.076
R1	0	0	8	7.0	
R2	0	0	0	0	
Peritoneal metastases (PM)					
Synchronous	4	9.3	25	22	0.076
Metachronous	5	13	25	28	0.069

*ASA* American Society of Anesthesiologists; *HIPEC* hyperthermic intraperitoneal chemotherapy, *PM* peritoneal metastases of colorectal origin, *R0* radical resection with >1-mm tumor-free margin, *R1* microscopically non-radical resection (≤1 mm margin), *R2* macroscopically non-radical resection

Baseline characteristics displayed for the subgroups: (1) peritoneal reaction with tumor less than 1 mm from the peritoneal surface (“<1 mm”) and (2) true peritoneal tumor penetration (“penetration”)

### Incidences of PM

**Fig. 1:**
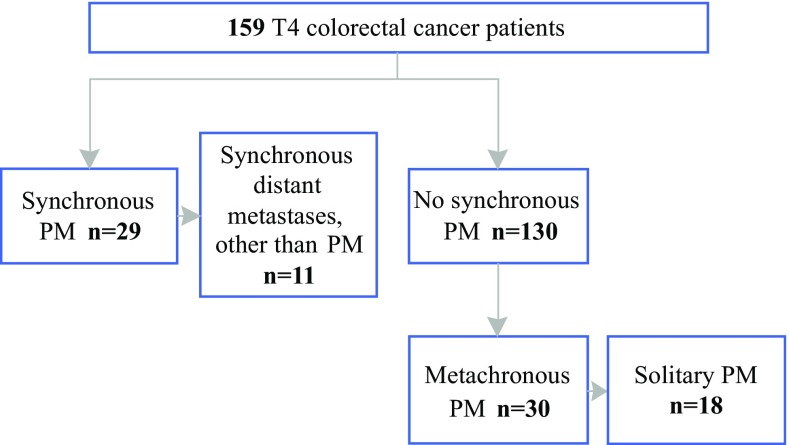
Incidences of peritoneal metastases (PM) of colorectal origin

Of the 159 patients, 29 (18%) had PM at the time the primary tumor (synchronous PM) was diagnosed (Fig. 1). All these patients underwent cytoreductive surgery (CRS), 10 of whom had surgery combined with HIPEC. Altogether, 18 patients had solitary PM, and 11 of the 29 patients also had other distant metastases diagnosed (10 with liver metastasis and 1 with lung and distant nodal metastases) at the time the primary tumor was diagnosed. Of the 130 patients without synchronous PM, 30 (23%) experienced metachronous PM during the follow-up period. The median time to diagnosis of metachronous PM was 17 months (IQR, 8–27 months), and 90% of metachronous PM was detected within 3 years. Of the patients who had peritoneal reaction with tumor less than 1 mm from the peritoneal surface, 4 (9.3%) presented with synchronous PM, and 5 (13%) experienced metachronous PM. Of the patients with true peritoneal tumor penetration, 25 (22%) presented with synchronous PM, and 25 (28%) experienced metachronous PM.

### Risk Factors for Synchronous and/or Metachronous PM

The results of logistic regression are displayed in Table 2. For this analysis, the PM status of 16 patients was categorized as “unknown” because they were lost to follow-up evaluation within 3 years with no signs of PM. Of the remaining 143 patients, 59 (41%) had PM diagnosed at some point in the course of their disease (synchronous/metachronous), and 84 (59%) did not experience PM during their follow-up period of at least 3 years or until death.

**Table 2:** Factors associated with peritoneal metastases (PM) of colorectal origin at any point in time (synchronous/metachronous)

	Univariable		Multivariable	
	OR (95% CI)	*p* value	OR (95% CI)	*p* value
Male gender (ref: female)	0.491 (0.249–0.970)	0.041*	0.531 (0.256–1.104)	0.090
Age (years) (ref < 60)		0.226		
60–70	0.864 (0.342–2.180)			
70–80	0.696 (0.271–1.784)			
>80	0.348 (0.120–1.006)			
Peritoneal penetration (ref: <1 mm)	2.929 (1.264– 6.787)	0.012*	2.518 (1.038–6.111)	0.041*
Grade (ref: well differentiated)		0.664		
Moderately	0.789 (0.211–2.951)			
Un/poorly Diff	0.618 (0.184–2.072)			
Mucinous component				
Partially/yes	1.754 (0.835–3.688)	0.138		
N stage (ref: N0)		0.002*		
*N* = 1	1.827 (0.787–4.240)		1.572 (0.651–3.797)	0.315
*N* = 2	5.220 (2.105–12.943)		4.046 (1.549–10.569)	0.004*
Synchronous distant metastases, other than PM	2.019 (0.934–4.365)	0.074*	1.431 (0.606–3.378)	0.413
Rectum (ref: colon)	1.463 (0.404–5.299)	0.562		
Left-side location of tumor (ref: right)	0.701 (0.352–1.397)	0.313		
Emergency surgery (ref: elective)	1.032 (0.469–2.271)	0.938		
R1 resection (ref: R0)	1.473 (0.287–7.566)	0.643		

Peritoneal penetration: true peritoneal tumor penetration

*Statistically significant

*OR* odds ratio; *CI* confidence interval, <1 mm, peritoneal reaction with tumor less than 1 mm from the peritoneal surface; *R1* microscopically non-radical resection (≤1-mm margin); *R0* radical resection with >1-mm tumor-free margin

In the univariable regression analysis, female gender, true peritoneal penetration, lymph node involvement, and synchronous distant metastases other than PM were significantly associated with PM. These variables were included in a multivariable analysis, with true peritoneal penetration (odds ratio [OR], 2.518; 95% confidence interval [CI], 1.038–6.111; *p* = 0.041) and lymph node involvement (N1: OR, 1.572; 95% CI, 0.651–3.797 vs N2: OR, 4.046; 95% CI, 1.549–10.569; *p* = 0.014) remaining significantly associated with PM.

### Risk factors for Metachronous PM

Table S3 presents the results of the Cox regression analysis for development of metachronous PM in the subgroup of 130 patients without synchronous PM. In the univariable analysis, gender, peritoneal involvement, lymph node involvement, and synchronous distant metastases other than PM were significant predictive factors. None of these variables remained significantly associated with metachronous PM in the multivariable analysis.

**Fig. 2:**
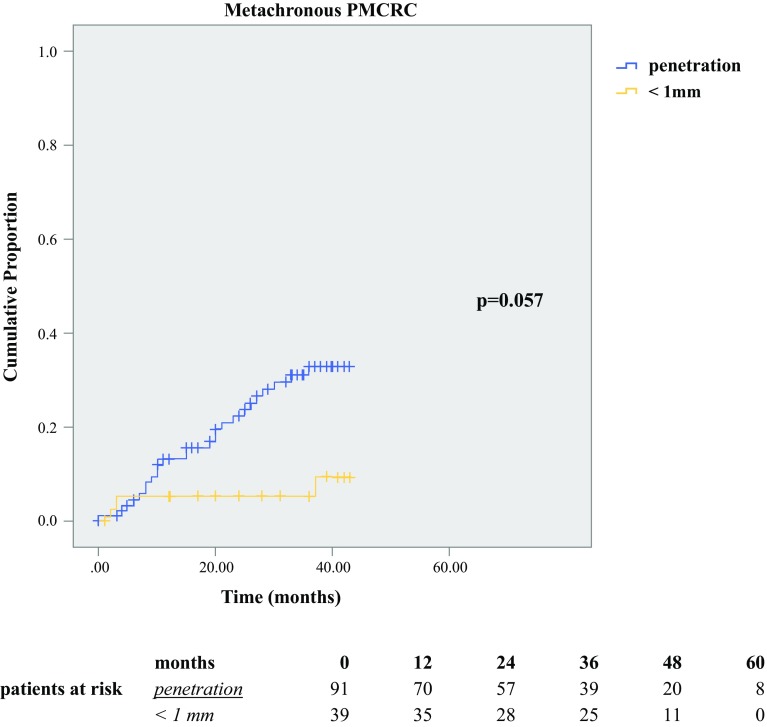
Metachronous peritoneal metastases (PM) of colorectal origin: the effect of peritoneal involvement. Development of metachronous PM in patients who had T4 colorectal cancer with (1) true peritoneal tumor penetration (“penetration”) and (2) peritoneal reaction with tumor less than 1 mm from the peritoneal surface (“<1 mm”) (*p* = 0.057, log-rank). Truncation at 43 months, when the patients at risk became less than one-third of the starting group

**Fig. 3:**
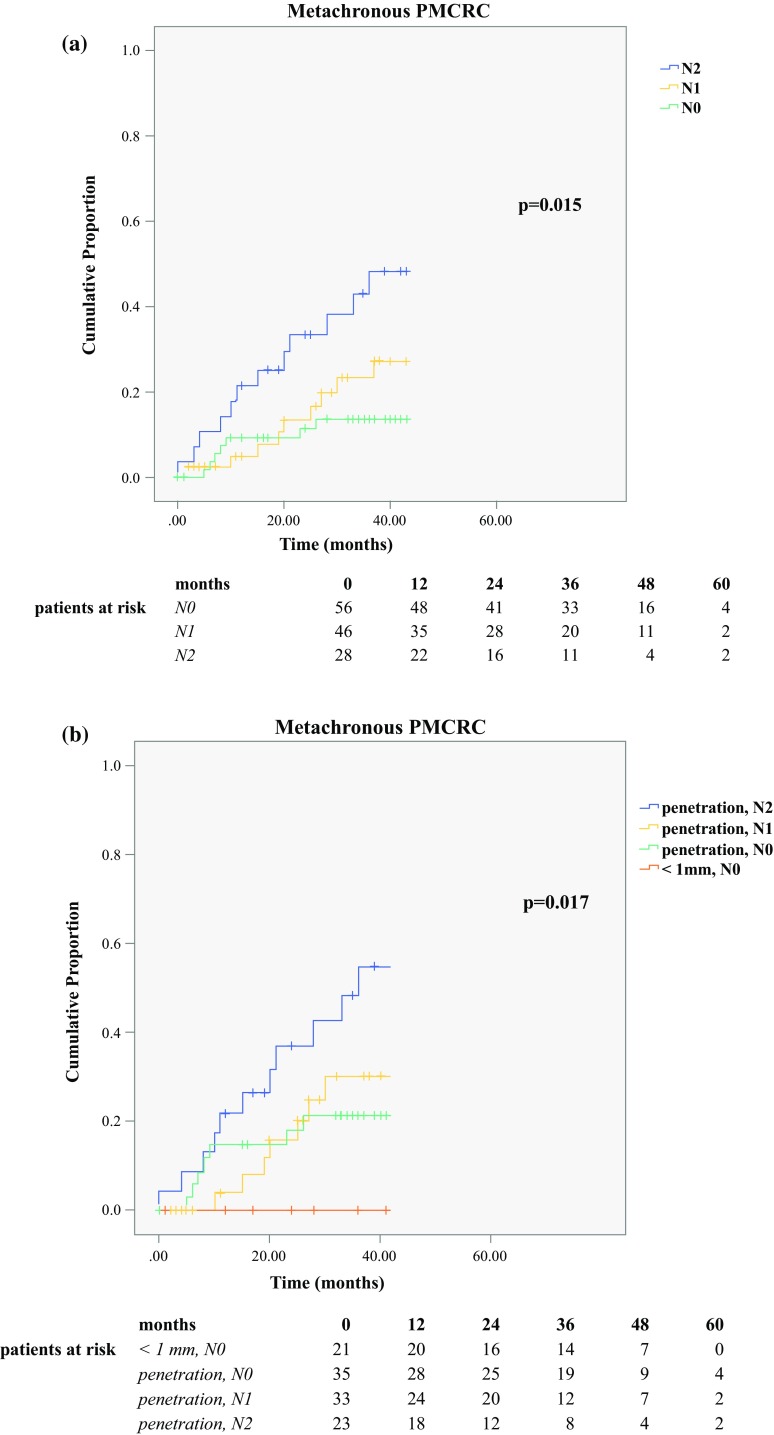
Metachronous peritoneal metastases (PM) of colorectal origin. **a** The effect of N stage. Development of metachronous PM in patients with T4 colorectal cancer in stages N0, N1, and N2 (*p* = 0.015, log-rank). Truncation at 43 months, when the patients at risk became less than one-third of the starting group. **b** Combined effect of N stage and peritoneal involvement. Development of metachronous PM in patients who had T4 colorectal cancer with (1) true peritoneal tumor penetration and N2 stage disease (“penetration, N2”), (2) true peritoneal tumor penetration and N1 stage disease (“penetration, N1”), (3) true peritoneal tumor penetration and N0 stage disease (“penetration, N0”), and (4) peritoneal reaction with tumor less than 1 mm from the peritoneal surface and N0 stage disease (“<1 mm, N0”) (*p* = 0.017, log-rank). Truncation at 42 months, when the patients at risk became less than one-third of the starting group

In Fig. 2, the Kaplan–Meier curve shows the proportion of PM over time for the two histologic subgroups regarding peritoneal involvement. For true peritoneal penetration, the 5-year PM proportion is 33%, as opposed to 21% for peritoneal reaction with tumor less than 1 mm from the peritoneum (*p* = 0.057). Also Kaplan–Meier curves showing the development of PM for subgroups based on lymph node status are displayed (Fig. 3a), showing 5-year PM proportions of 23% for N0-stage, 27% for N1-stage, and 48% for N2-stage disease (*p* = 0.015). Figure 3b shows the proportion of PM over time for the subgroups based on both peritoneal involvement and lymph node status.

Of the 159 CRC patients in this study, 104 were staged with T4NxM0 disease. For 99 of these patients, the tumor was resected radically (R0), and this subgroup could have been eligible for prophylactic therapy (i.e., adjuvant HIPEC) or second-look surgery. Metachronous PM developed for 22 (22%) of the 99 patients in this subgroup. In the univariable analysis, female gender, peritoneal penetration, and lymph node involvement were significantly associated with the development of PM. Peritoneal penetration did not remain significantly associated with PM in the multivariable analysis (hazard ratio [HR], 2.094; 95% CI, 0.697–6.287; *p* = 0.188), whereas N2 stage disease did remain significantly associated with PM (N2: HR, 3.134; 95% CI, 1.120–8.764; *p* = 0.030).

## Discussion

This study showed an overall risk of 37% for the development of PM at some point in the course of disease for CRC patients with tumors nearby or penetrating the peritoneum. True peritoneal penetration, in contrast to peritoneal reaction with tumor less than 1 mm from the peritoneum (systematically distinguished by dedicated pathologists), was significantly associated with the overall development of PM. Also, lymph node involvement was an independent risk factor for the development of PM in this study.

For metachronous PM, peritoneal penetration was shown to be a significant risk factor only in the univariable analysis. This might have been due to restricted statistical power, although this was one of the largest cohorts reported on this subject. The 5-year risk of metachronous PM was 33% for the patients with tumors that had true peritoneal penetration and 21% for the patients with tumors that had peritoneal reaction with tumor less than 1 mm from the peritoneum. Both proportions are substantially higher than the 5-year risk of 10% for patients with T3 colon tumors reported by Hompes et al.^13^ To explore very high-risk subgroups, lymph node status and peritoneal involvement were combined, showing a 55% 5-year risk of metachronous PM for the patients with true peritoneal tumor penetration and N2-stage disease.

In the current T4a/b staging systems, careful evaluation of the exact peritoneal involvement is not taken into account. In cases of T4b, the peritoneum (serosa) may not be involved (e.g., in retroperitoneally located tumors in the ascending or descending colon or in rectal tumors distal to the peritoneal fold). Also, the definitions have varied over time. According to TNM5, T4a is defined as “extension into nearby structures” and T4b as “perforation of the bowel.” With respect to perforation, no distinction between perforation of the tumor and bowel perforation proximal to an obstructive tumor is made. In the latter subgroup, peritoneal involvement of the tumor is mostly absent.

In the literature, the association between peritoneal penetration and the development of PM often is assessed using T4a/b subcategorization. A subgroup of T4a colorectal carcinomas (TNM7) has been shown to have a higher metachronous PM rate (50%, 7 of 14 patients) than a T4b subgroup (20%, 1 of 5 patients).^13^ However, another study with a larger number of T4 patients (*n* = 200) showed no difference in synchronous (23 vs. 24%) or metachronous (24 vs. 17%) PM between patients with T4a and T4b tumors,^14^ nor did other studies.^15^ To improve risk stratification, a more careful description of the extent of peritoneal involvement should be incorporated into our T4a/T4b staging system.

Panarelli et al.^16^ and Snaebjornsson et al.^17^ recently elucidated the controversy concerning the T4 definition from a pathologic perspective. Snaebjornsson et al.^17^ reassessed pathologic T-stage and the LPI score in a nationwide colon cancer cohort (*n* = 889) and confirmed the high prognostic impact of the pT4 stage, even over lymph node involvement. He brought the definition of T4 into discussion with regard to the LPI score. It remains controversial what LPI score should be regarded as T4. These authors showed that T4b tumors (TNM7) with true peritoneal penetration (LPI4) have the worst survival rate and should be regarded as a separate category. Also, they showed that the addition of LPI3 tumors to the T4a category clearly improves the survival rate for T4a patients. They did not find a survival difference between “true” T4a (T4a + LPI4, peritoneal penetration) and “true” T4b (T4b + LPI1-3, organ involvement without peritoneal penetration) tumors. However, these authors did not separately assess development of PM, for which the LPI score might have been of particular value.

Lymph node involvement also was identified as an independent risk factor for PM in this study. This finding was confirmed in the literature.^2^,^3^,^5^,^14^ However, considering metachronous PM, the value of lymph node involvement as an independent risk factor remains controversial.^2^–^4^,^14^,^18^ Also in this study, lymph node involvement was significantly associated with metachronous PM only in the univariable analysis, and lost its significance in the multivariable analysis due to the smaller sample in this sub-analysis.

Kaplan–Meier curves (Fig. 3a) show that proportions of PM increase with N stage, reaching the highest proportion (49%) in N2-stage patients (HR, 3.206; 95% CI, 1.348–7.627; *p* = 0.008). These findings suggest an association between lymph node positivity and peritoneal metastases. It could be hypothesized that dissection of lymphatic vessels during colectomy could lead to an intraperitoneal tumor spill. Another explanation could be the existence of a CRC subtype with a high metastatic potential, causing both lymph node and peritoneal metastases.

Both adjuvant HIPEC and CRS/HIPEC are applied only when other distant metastases have not developed, or in case of limited resectable liver metastases. Other distant metastases also were synchronously diagnosed for 38% (*n* = 11) of the patients with synchronous PM in this study (Fig. 1), compromising their eligibility for CRS/HIPEC. For 40% (*n* = 12) of the patients presenting with metachronous PM, the peritoneum was the only affected site. This subgroup in particular might benefit from the new treatment strategies.

The patient database of this study was prospectively kept with systematic scoring of the peritoneal involvement by the pathologists. However, other variables and long-term disease outcomes were retrospectively collected. The restricted sensitivity of imaging methods for the small peritoneal nodules,^19^,^20^ together with the retrospective character of this study, probably resulted in an underestimation of PM incidences. Also, because the UZ Leuven is a tertiary referral center, the study population reflected a selected (high-risk) patient group, and some patients were lost to follow-up evaluation because they were followed up in the referring hospital. Furthermore, although selection of blocks and slides from the colorectal cancer specimen during the pathologist’s workup has been standardized, collection of “sufficient” material remains arbitrary to some extent. True peritoneal tumor penetration might have been missed in some of the cases that had peritoneal reaction with tumor less than 1 mm from the tumor.

In conclusion, histologic confirmation of true peritoneal tumor penetration by CRC constitutes a high-risk subset of tumors regarding the development of PM. Based on this finding, incorporating evaluation of the exact peritoneal tumor involvement in the tumor-node-metastasis (TNM) classification should be considered. For the patients with true peritoneal tumor penetration and N2 stage, a 5-year risk of 55% for metachronous PM was found in this study. This group in particular might be eligible for new treatment strategies that aim to treat PM in an earlier or even preventive setting. However, the results of trials investigating these strategies should be awaited.

## Electronic Supplementary Material

Below is the link to the electronic supplementary material.

Supplementary material 1 (DOC 182 kb)
