# Supplementary material for: Locally Advanced Colorectal Cancer: True Peritoneal Tumor Penetration is Associated with Peritoneal Metastases
Source: Ann Surg Oncol. 2017 Oct 26;25(1):212–20. doi: 10.1245/s10434-017-6037-6 (PMC5740196; doi:10.1245/s10434-017-6037-6)
Supplement: Supplementary file 1 — Supplementary material 1 (DOC 182 kb) [file 10434_2017_6037_MOESM1_ESM.doc]

| **Suppl. table 1. TNM 7** | |
| --- | --- |
| **T - primary tumor** | |
| T0 | No evidence of primary tumor |
| T1 | Tumor invades submucosa |
| T2 | Tumor invades muscularis propria |
| T3 | Tumor invades subserosa or into non-peritonealized pericolic or perirectal tissues |
| T4 | Tumor directly invades other organs or structures and/or perforates visceral peritoneum |
|  | T4a: Tumor perforates visceral peritoneum |
|  | T4b: Tumor directly invades other organs or structures^1,2^ |
| *Note 1* | Direct invasion in T4b includes invasion of other organs or segments of the colorectum by way of the serosa, as confirmed on microscopic examination, or for tumors in a retroperitoneal or subperitoneal location, direct invasion of other organs or structures by virtue of extension beyond the muscularis propria. |
| *Note 2* | Tumor that is adherent to other organs or structures, macroscopically, is classified cT4b. However, if no tumor is present in the adhesion, microscopically, the classification should be pT1-3, depending on the anatomical depth of wall invasion. |

| **Suppl. table 2. Local Peritoneal Involvement (LPI) score according to Shepherd**^8^ | |
| --- | --- |
| **LPI - primary tumor** | |
| LPI1 | Tumor well clear of closest peritoneal surface |
| LPI2 | Mesothelial inflammatory and/or hyperplastic reaction with tumor close to, but not at the peritoneal surface |
| LPI3 | Tumor present at peritoneal surface with inflammatory reaction, mesothelial hyperplasia and/or erosion or ‘ulceration’ |
| LPI4 | Tumor cells shown free on the serosal surface with evidence of adjacent ‘ulceration’ of the visceral peritoneum. |

| **Suppl. table 3. Risk factors for metachronous PM** | | |  |  |  | |
| --- | --- | --- | --- | --- | --- | --- |
|  |  | Univariable | | Multivariable | | |
|  |  | **Hazard ratio** | p-value | HR | | p-value |
| Male gender (ref: female) | | 0.458 (0.210-1.000) | 0.050* | 0.519 (0.235-1.146) | | 0.105 |
| Age (ref <60) |  |  | 0.682 |  | |  |
|  | 60-70 | 0.708 (0.256-1.953) |  |  | |  |
|  | 70-80 | 0.871 (0.331-2.292) |  |  | |  |
|  | >80 | 0.513 (0.163-1.621) |  |  | |  |
| Peritoneal penetration (ref: tumor <1mm) | | 2.465 (0.942-6.450) | 0.066* | 1.932 (0.718-5.203) | | 0.192 |
| Grade (ref: well differentiated) | |  | 0.518 |  | |  |
|  | moderately | 3.389 (0.416-27.600) |  |  | |  |
|  | un-/poorly diff | 3.054 (0.410-22.718) |  |  | |  |
| Mucinous component | partially / yes | 1.019 (0.437-2.377) | 0.965 |  | |  |
| N-stage (ref: N0) | |  | 0.022* |  | |  |
|  | N=1 | 1.400 (0.555-3.529) |  | 1.278 (0.503-3.246) | | 0.606 |
|  | N=2 | 3.206(1.348-7.627) |  | 2.332 (0.912-5.966) | | 0.077 |
| Synchronous distant metastases, other than PM | | 2.037 (0.904-4.588) | 0.086* | 1.354 (0.567-3.320) | | 0.495 |
| Colon (ref: rectum) | | 1.805 (0.545-5.980) | 0.334 |  | |  |
| Left sided location of tumor (ref: right) | | 0.804 (0.384-1.684) | 0.563 |  | |  |
| Emergency surgery | | 0.920 (0.394-2.147) | 0.848 |  | |  |
| Adjuvant therapy | | 0.843 (0.562-1.264) | 0.408 |  | |  |
| Perforation (clinical/peroperative) | | 0.930(0.281-3.076) | 0.905 |  | |  |
| Radicality of resection | | 1.278 (0.173-9.458) | 0.810 |  | |  |

*Suppl. table 3. Risk factors for metachronous PM.* Peritoneal penetration: true peritoneal tumor penetration; PM: peritoneal metastases of colorectal origin. R0: radical resection with >1mm tumor-free margin, R1: microscopically non-radical resection (≤ 1mm margin); <1mm: peritoneal reaction with tumor within 1 mm of the peritoneal surface.

*Suppl. figure 1. Included patients* CRC: colorectal carcinoma
